# Epigenome‐wide Association of DNA Methylation in Whole Blood With Bone Mineral Density

**DOI:** 10.1002/jbmr.3148

**Published:** 2017-05-08

**Authors:** John A Morris, Pei‐Chien Tsai, Roby Joehanes, Jie Zheng, Katerina Trajanoska, Mette Soerensen, Vincenzo Forgetta, Juan Edgar Castillo‐Fernandez, Morten Frost, Tim D Spector, Kaare Christensen, Lene Christiansen, Fernando Rivadeneira, Jonathan H Tobias, David M Evans, Douglas P Kiel, Yi‐Hsiang Hsu, J Brent Richards, Jordana T Bell

**Affiliations:** ^1^ Department of Human Genetics McGill University Montreal Canada; ^2^ Lady Davis Institute for Medical Research Department of Medicine Jewish General Hospital McGill University Montreal Canada; ^3^ Department of Twin Research and Genetic Epidemiology King's College London London UK; ^4^ Institute for Aging Research Hebrew SeniorLife Boston MA USA; ^5^ Department of Medicine Beth Israel Deaconess Medical Center and Harvard Medical School Boston MA USA; ^6^ MRC Integrative Epidemiology Unit University of Bristol Bristol UK; ^7^ Departments of Internal Medicine and Epidemiology Erasmus Medical Center Rotterdam Netherlands; ^8^ The Danish Twin Registry and The Danish Aging Research Center Epidemiology, Biostatistics, and Biodemography Institute of Public Health University of Southern Denmark Odense Denmark; ^9^ Department of Clinical Genetics Odense University Hospital Odense Denmark; ^10^ Endocrine Research Unit KMEB University of Southern Denmark Odense Denmark; ^11^ Department of Clinical Biochemistry and Pharmacology Odense University Hospital Odense Denmark; ^12^ Musculoskeletal Research Unit University of Bristol Bristol UK; ^13^ The University of Queensland Diamantina Institute Translational Research Institute Princess Alexandra Hospital Brisbane Australia; ^14^ Broad Institute of MIT and Harvard Cambridge MA USA; ^15^ Departments of Epidemiology and Biostatistics and Medicine McGill University Montreal Canada

**Keywords:** BONE MINERAL DENSITY, EPIGENETICS, DNA METHYLATION, EPIGENOME‐WIDE ASSOCIATION STUDY (EWAS), GENETIC EPIDEMIOLOGY

## Abstract

Genetic and environmental determinants of skeletal phenotypes such as bone mineral density (BMD) may converge through the epigenome, providing a tool to better understand osteoporosis pathophysiology. Because the epigenetics of BMD have been largely unexplored in humans, we performed an epigenome‐wide association study (EWAS) of BMD. We undertook a large‐scale BMD EWAS using the Infinium HumanMethylation450 array to measure site‐specific DNA methylation in up to 5515 European‐descent individuals (*N_Discovery _*= 4614, *N_Validation _*= 901). We associated methylation at multiple cytosine‐phosphate‐guanine (CpG) sites with dual‐energy X‐ray absorptiometry (DXA)‐derived femoral neck and lumbar spine BMD. We performed sex‐combined and stratified analyses, controlling for age, weight, smoking status, estimated white blood cell proportions, and random effects for relatedness and batch effects. A 5% false‐discovery rate was used to identify CpGs associated with BMD. We identified one CpG site, cg23196985, significantly associated with femoral neck BMD in 3232 females (*p *= 7.9 ×* *10^−11^) and 4614 females and males (*p *= 3.0 × 10^−8^). cg23196985 was not associated with femoral neck BMD in an additional sample of 474 females (*p *= 0.64) and 901 males and females (*p *= 0.60). Lack of strong consistent association signal indicates that among the tested probes, no large‐effect epigenetic changes in whole blood associated with BMD, suggesting future epigenomic studies of musculoskeletal traits measure DNA methylation in a different tissue with extended genome coverage. © 2017 The Authors. *Journal of Bone and Mineral Research* Published by Wiley Periodicals Inc.

## Introduction

Osteoporosis is primarily an aging‐related disease characterized by compromised bone strength that increases the risk of fracture. Because of population aging worldwide, the incidence of osteoporosis is increasing, exceeding $17 billion per year in direct care costs within the United States^1^ and costing upward of €37 billion per year in the EU‐27 member states.^2^ Identifying the causes of osteoporosis will improve the understanding of its pathology, leading to better or more efficient treatments of this common and costly disease. Low bone mineral density (BMD) is one of the major risk factors for fracture and is largely used in clinical prediction tools for fracture and gauging response to treatment. Genome‐wide association studies (GWAS) of BMD directly assessed at the femoral neck (FN) and lumbar spine (LS), the two most commonly measured sites for quantifying BMD and diagnosing osteoporosis, have been instrumental in identifying novel genetic loci influencing osteoporosis disease risk.^3^, ^4^ However, epigenetic variation in the genome, which can be influenced by both genetic and environmental factors,^5^, ^6^ may also influence BMD, yet the epigenetic influences on BMD have largely been unexplored.

One of the most stable epigenetic processes is DNA methylation, or the addition of a CH_3_ methyl group to cytosine, typically in the context of cytosine paired sequentially to a guanine nucleotide, separated by a phosphate group (CpG). DNA methylation is known to play a role in gene expression and cell differentiation,^7^, ^8^ and differential DNA methylation has been linked to multiple human complex traits and disease phenotypes.^5^, ^9^, ^10^, ^11^, ^12^ Studies performed using bone samples have identified epigenetic alterations that influence bone cell function.^13^, ^14^ We studied epigenetic variation in whole blood, as a proxy for difficult‐to‐acquire samples such as bone, in relation to BMD because epigenetic markers are often stable across multiple tissues, and immune cells within blood are known to influence bone homeostasis.^15^ Furthermore, osteoclasts are derived from the monocyte‐macrophage lineage found in whole blood.^16^ Although epigenetic profiling has been performed previously in bone samples from osteoporotic and osteoarthritic patients^17^ and an epigenome‐wide association study (EWAS) of BMD has been performed in mice,^18^ EWAS of BMD have not been reported in humans with validation of significant findings.

We, therefore, undertook a large‐scale BMD EWAS, assessing the association of up to 473,882 CpGs quantified in whole blood with BMD measured in up to 4614 individuals across five cohorts from Europe and North America. To our knowledge, this study is the largest EWAS of a musculoskeletal trait performed to date. We used an additional 901 individuals as a validation cohort to increase the reliability of our results.

## Materials and Methods

### Individual cohorts

We performed our EWAS in cohorts composed of European‐descent individuals. Cohorts used for the discovery analysis were the TwinsUK Registry (TUK), Framingham Study Offspring Cohort (FOS), Avon Longitudinal Study of Parents and Children (ALSPAC; further information on the ALSPAC cohorts and ARIES project is included in Supplemental Table S1), Rotterdam Study (RS), and the Danish Twin Registry (DTR). The cohort used for validation of significant findings was the Framingham Study Generation 3 cohort (Gen3), a cohort including family members of FOS (Table 1; Supplemental Table S1).

**Table 1:** Sample Sizes of Discovery and Replication Cohorts

		Sample size					
		FN BMD			LS BMD		
Phase	Cohort	Pooled	Females	Males	Pooled	Females	Males
	Avon Longitudinal Study of Parents and Children (ALSPAC)	715	715	0	0	0	0
	Danish Twin Registry (DTR)	267	132	135	260	132	128
Discovery	Framingham Study Offspring Cohort (FOS)	2207	1254	953	2203	1259	953
	Rotterdam Study (RS)	650	356	294	633	346	287
	TwinsUK (TUK)	775	775	0	770	770	0
	Discovery total	4614	3232	1382	3866	2507	1368
Validation	FOS Gen3	901	448	453	0	0	0
	Discovery + Validation total	5515	3680	1835	3866	2507	1368

FN = femoral neck; BMD = bone mineral density; LS = lumbar spine.

Both, or one of, FN and LS BMD were measured in each cohort by dual‐energy X‐ray absorptiometry (DXA) (Supplemental Table S2). All cohorts, except the ALSPAC and DTR, followed the same methods for extracting DNA from whole‐blood tissue and quantifying DNA methylation. Whole‐blood tissue DNA was extracted using the DNeasy Blood & Tissue Kit (Qiagen, Inc., Valencia, CA, USA), followed by bisulfite conversion of 750 ng DNA using the EZ DNA Methylation Kit (Zymo Research, Irvine, CA, USA) following manufacturer instructions. The ALSPAC cohort and the DTR cohort performed DNA extraction and conversion as described previously.^19^, ^20^ DNA methylation across the genome was quantified using the Infinium HumanMethylation450 BeadChip (Illumina, San Diego, CA, USA), assaying up to 482,421 CpGs throughout the human genome. Image intensities were extracted using GenomeStudio Methylation Module (v1.8) software. Cohort‐specific criteria were applied in further quality control and normalization of probe intensities (Supplemental Table S3).

### Statistical analyses

For discovery analyses, each cohort followed a prespecified analysis plan. FN and LS BMD residuals were calculated by fitting a linear regression model, adjusting for age, weight, and sex. For sex‐specific analyses, the term for sex was removed from the model. In addition, the DTR adjusted for birth weight discordance as historically DTR samples were selected to address birth weight discordance in twins (Supplemental Table S1). To address the issue of cell heterogeneity in whole blood tissue, each cohort calculated the estimated white blood cell proportions of B cells, T cells (CD4+ and CD8+), granulocytes, natural killer cells, and monocytes using the Houseman and colleagues method for quadratic projections.^21^ DNA methylation for each probe was transformed to a standard normal distribution using quantile normalization. The association between DNA methylation and BMD was then calculated by fitting a linear mixed effects model for normalized DNA methylation, including BMD residuals, smoking (measured as smokers, non‐smokers, or former smokers), age, weight, sex, and estimated white blood cell proportions as fixed effects, and terms for family structure and batch effects as random effects, where relevant. We used BMD residuals to reduce problems due to collinearity between BMD, weight, and age when fitting our linear mixed‐effects model. Association testing was performed in male, female, and combined samples. Each cohort was assessed for epigenome‐wide statistical inflation by calculating the genomic inflation factor lambda (*λ*) and generating a quantile‐quantile plot (QQ‐plot). Lambda can be calculated to estimate the deviation of a distribution from a null expected distribution, whereas QQ plots can be used to visualize the deviation of a distribution from a null expected distribution.

Fixed‐effects meta‐analyses were performed using METAL^22^ for FN and LS sex‐combined and sex‐stratified analyses. We used the *I^2^* statistic to quantify the variability in association effect estimates due to statistical heterogeneity, excluding probes with heterogeneous *I^2^* statistics (*p_Het_* < 0.05). Statistical significance, when considering the multiple testing burden, was determined by calculating Benjamini‐Hochberg (BH) adjusted *p* values for each meta‐analysis. Probes with significant BH‐adjusted *p* values (*p_BH_* < 0.05) would, therefore, be significant at a 5% false‐discovery rate (FDR). Summary statistics for the FN and LS sex‐combined and sex‐stratified meta‐analyses are available for download (www.gefos.org).

Associated probes were assessed for their twin‐based heritability using normalized methylation beta values estimated from 330 female MZ twin pairs and 34 female DZ twin pairs from the TUK cohort, adjusted for age, body mass index, smoking status, alcohol consumption, predicted whole blood cell‐type counts, DNA methylation plate, and position on the plate. Heritability was estimated by fitting the classical ACE model in OpenMX.^23^ The observed variance in the adjusted beta values was partitioned into additive genetic (A), common environmental (C), and unique environmental (E) factors. Heritability was defined as A/(A+C+E).

Associated probes were assessed for the influence of single nucleotide polymorphisms (SNPs) that overlapped the probe body by mapping these probes to dbSNP 146.^24^ This was assessed by adding a term for the dosage of each SNP to the discovery linear mixed‐effects models in cohorts with genotype data to observe if the association between DNA methylation and BMD was influenced by the genetic polymorphism at the probe.

Gen3 samples were assessed using the same methods as in the analysis of FOS samples to perform validation analyses of significantly associated probes. Probes were deemed robustly associated with BMD if they met a validation *p* value of less than 0.05. These samples are not completely independent from the FOS samples because the Framingham Study is a family‐based study with several cohorts, and, therefore, there is underlying family structure.

To assess the power of our study, we performed 5000 permutations on the 775 TUK samples with FN BMD measurements. FN was randomly sampled based on the twin and family structure before fitting linear mixed‐effects models, and the power was defined as the number of permutations with *p* values greater than the observed *p* value for the TUK samples (*p* = 1.14 × 10^−5^; Supplemental Table S8).

## Results

### Meta‐analysis

**Figure 1:**
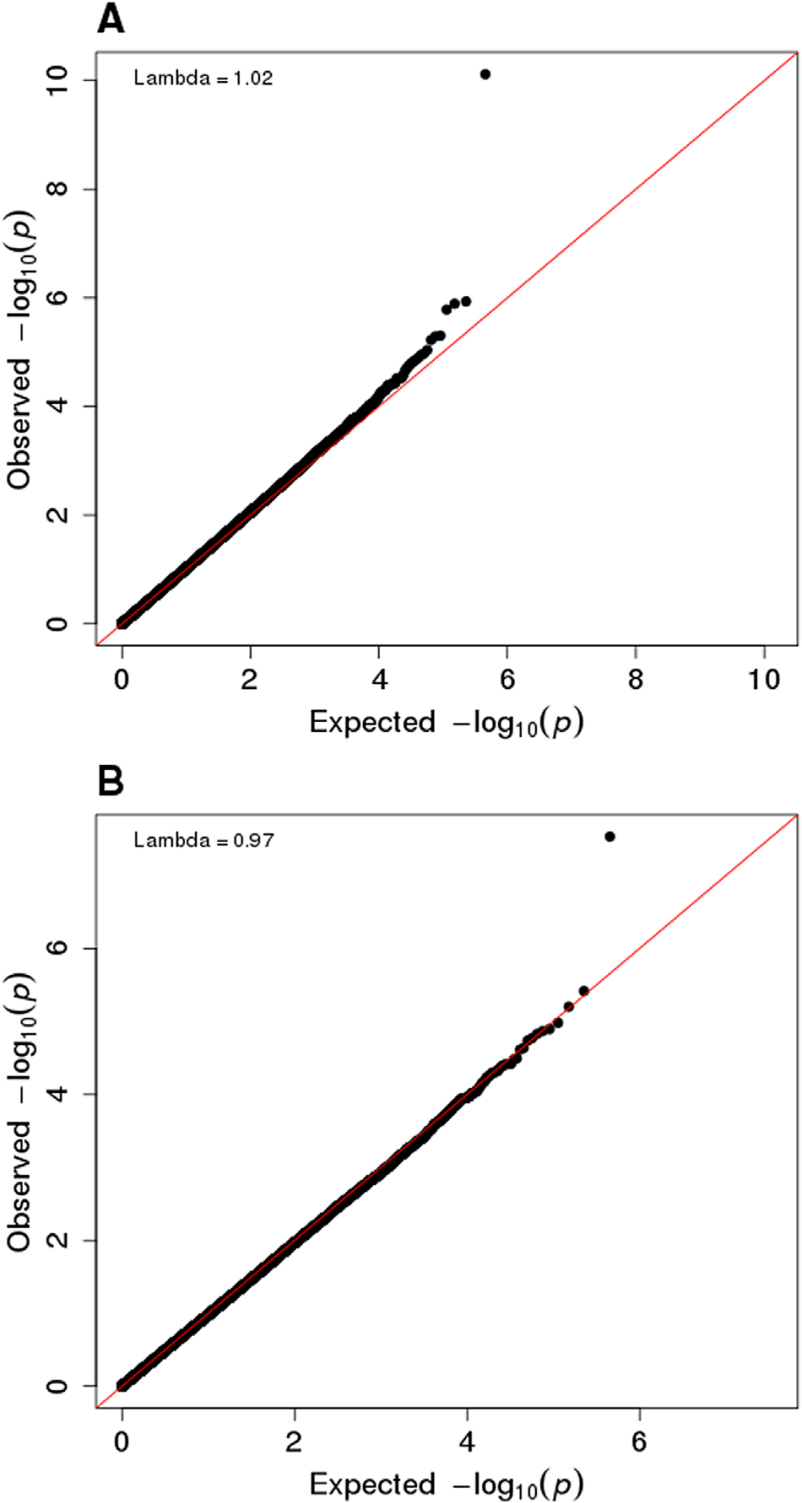
Quantile‐quantile plots (QQ plots) of the distribution of observed −log10 association *p* values against the expected null distribution, for discovery meta‐analyses of FN BMD in (*A*) females‐only and (*B*) sex‐combined analyses. Genomic inflation lambda scores are given in each QQ plot to quantify statistical inflation of *p* values. No evidence for inflation was observed in the QQ plots or as calculated by lambda scores.

**Figure 2:**
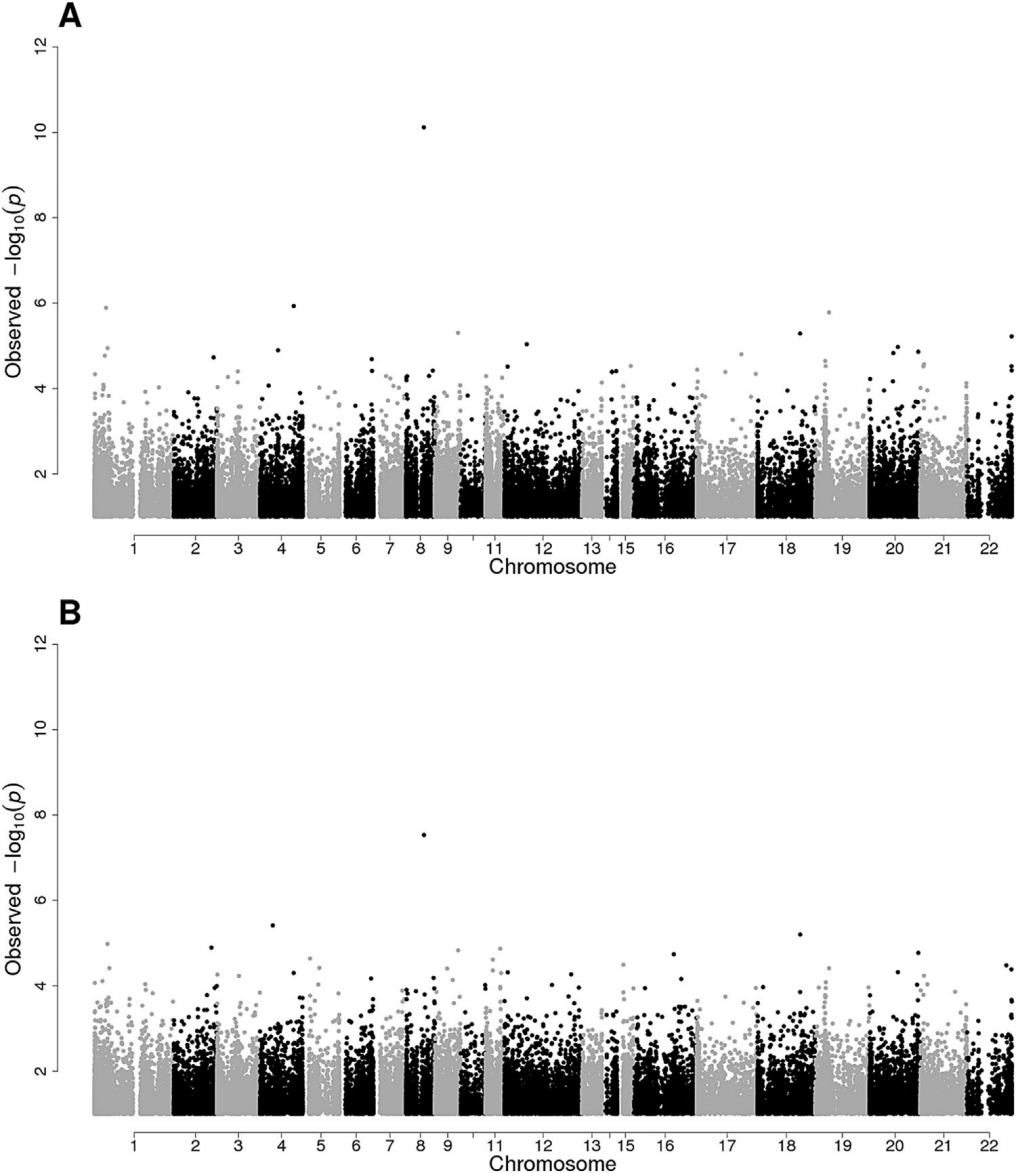
Manhattan plots of −log_10_ association *p* values for discovery meta‐analyses of FN BMD in (*A*) females‐only and (*B*) sex‐combined analyses.

Meta‐analyses of discovery cohorts identified CpG site cg23196985 associated at a 5% FDR for FN sex‐combined (*β* = 0.66, SE = 0.19, *p* = 2.99 × 10^−8^, *p_BH_* = 1.30 × 10^−2^) and FN female (*β* = 0.95, SE = 0.15, *p* = 7.86 × 10^−11^, *p_BH_* = 3.41 × 10^−5^; Fig. 1, Fig. 2, Table 2) analyses. CpG site cg23196985 maps to the 5’ untranslated region of the liver carboxylase 1 gene (*CES1*), which is expressed in the liver and whole blood^25^ yet with no currently reported associations with BMD by GWAS in the same chromosomal region (16q12.2) and with the nearest BMD‐associated SNP mapping approximately 4 mega base pairs upstream at the *SALL1* and *CYLD* locus.^26^ The calculated lambdas and QQ plots for the meta‐analyses of FN female and sex‐combined analyses revealed no statistical inflation of the association *p* values (*λ*_female_ = 1.02, *λ*_sex‐combined_ = 0.97; Supplemental Table S9). We observed no significantly associated CpG sites with LS BMD in sex‐combined or sex‐stratified analyses (Supplemental Figures).

**Table 2:** Meta‐Analysis Results for Association of cg23196985 with FN BMD in Both Female and Sex‐Pooled Analyses in Discovery phase, with Replication and Combined Discovery and Replication Analyses

	Discovery					Validation			Combined			
	*β*	SE	*p*	*p_BH_*	*p_Het_*	*β*	SE	*p*	*β*	SE	*p*	*p_Het_*
Female	0.95	0.15	7.9E‐11	3.4E‐05	0.81	0.19	0.39	0.64	0.86	0.14	3.8E‐10	0.43
Sex pooled	0.66	0.12	3.0E‐08	1.3E‐02	0.1	–0.01	0.02	0.6	0.01	0.02	0.68	3.0E‐07

FN = femoral neck; BMD = bone mineral density; *β* = effect size; SE = standard error; *p_BH_* = Benjamini‐Hochberg adjusted *p* value; *p_Het_* = heterogeneity *p* value.

We tested for the influence of SNPs underlying cg23196985 in females from the FOS, RS, and ALSPAC cohorts, as the strength of the association was stronger in females than in the sex‐combined analysis. Because some of the samples in our cohorts included twins, we first estimated the evidence for heritability of DNA methylation levels at cg23196985 in the TUK cohort. We observed evidence for additive genetic effects with a heritability estimate of 0.69 at cg23196985 and therefore pursued further analyses exploring the association between DNA methylation levels at this CpG site and BMD conditional on SNP genotypes. All twins were homozygous for the reference allele at rs144950224, a SNP that maps directly to the probe's target CpG site. Four SNPs mapped to the cg23196985 50 base‐pair probe sequence, and these included rs144950224, rs12149371, rs12149373, and rs3815583. SNP rs144950224 was found to be rare within our cohorts, with a minor allele frequency (MAF) of approximately 0.5% in FOS samples, 0.1% in RS samples, and no carriers in ALSPAC samples. We observed no notable change in association *p* values upon conditioning with each of the four SNPs (Supplemental Table S10).

In the validation sample, cg23196985 was not associated with FN in Gen3 female (*p* = 0.64) and sex‐combined (*p* = 0.60) analyses. However, after meta‐analyzing Gen3 validation data with discovery results, the probe remained strongly associated in female‐only analyses (*β* = 0.86, SE = 0.14, *p* = 3.7 × 10^−10^; *n* = 4345) but not in sex‐combined analysis (*p* = 0.68, *n *= 5301).

For our power calculation, we found all permutations were less significant than our observed *p* value for the 775 TUK samples at *p* = 1.14 × 10^−5^ (permuted *p* value range 0.99 to 2.46 × 10^−5^); this suggested we had 100% power to detect the observed effect size (*β* = 1.20, SE = 0.27) between bone density measurements and methylation at cg23196985 in *CES1*.

### Individual cohorts

Individual cohort analyses identified seven probes that were significantly associated in two cohorts with sex‐combined or sex‐stratified analyses (Supplemental Table S4), but there were no other cohort‐specific significant associations. The DTR LS female analysis identified four significantly associated probes, two of which map to genes, cg04081651 (*MAP3K8*), cg09832237, cg14793931 (*ZFR2*), and cg24029028 (Supplemental Table S5). The DTR LS male analysis identified one significantly associated probe, cg23214071 (*HLA*‐*DQB1*) (Supplemental Table S6). The TUK LS female analysis identified two significantly associated probes, cg24117468 (*P4HA2*) and cg02526790 (*TG*) (Supplemental Table S7). The calculated lambda and QQ plot for the DTR LS female analysis revealed statistical inflation of the association *p* values (*λ *= 1.46), but the lambda and QQ plots for the remainder of the cohorts showed no large inflation or deflation of association *p* values (Supplemental Table S9).

## Discussion

In the first large‐scale assessment of the contribution of epigenetic changes in whole blood to BMD, we did not identify methylation changes reliably associated with this clinically relevant trait. CpG site cg23196985 was found in the discovery meta‐analysis to be strongly associated with FN BMD in females only and in analyses combining males and females, but upon validation in an extended sample that included related individuals, the association was attenuated in the female analysis and completely absent in sex‐combined analyses.

These findings provide important insights into the field of epigenetics. The first is that by using a precisely measured trait, BMD, which is highly heritable with estimates from 50% to 85%^27^ and for which genetic determinants have been identified through GWAS,^3^, ^4^, ^27^ there do not appear to be associations between methylation changes and BMD. Although whole blood methylation changes may not be the ideal tissue within which to test epigenetic influences on bone, this conveniently accessible tissue has many links to bone biology, including the fact that osteoclasts and monocyte/macrophages originate from the same precursors.^15^, ^16^ The extent to which methylation changes are shared between bone and whole blood is not well known. However, evidence shows that a significant proportion of methylation variation genome‐wide can be conserved across tissues.^28^ Additional explanations for our mostly null findings include the possibility that DNA methylation changes may not have a large influence on BMD.

Notwithstanding the general lack of consistent associations with BMD across the genome, we did generate evidence for suggestive association of cg23196985 with FN BMD in females. However, we caution that these findings require further replication. Because we are unaware of any available replication data to test this hypothesis, these findings will require replication in future studies.

There is limited evidence for the effects of DNA methylation on bone. A methylation profiling study that compared the differences between bone samples of 27 osteoporotic and 23 osteoarthritic patients was undertaken on an earlier DNA methylation platform, the HumanMethylation27 BeadChip (assessing approximately 27,000 CpGs in the genome), and was able to identify bone genes following pathway analyses of more than 200 differentially methylated CpGs; however, to date, these results lack replication.^17^ Another study failed to demonstrate specific effects of DNA methylation, assessed by sequencing methods, on *RANKL* in the bones of patients with osteoporotic fractures.^29^ The evidence from previous studies and our own suggests that if strong effects of DNA methylation on bone biology are to be identified, they may not be detectable with current analytical approaches.

A strength of our study was the sample size and a conservative estimate of statistical power to identify epigenetic effects on BMD that account for 0.8% of its variance. The large sample size also allowed us to classify several cohort‐level associations that were likely to be false positives. For example, TUK female analyses identified two probes significantly associated with LS BMD, but this association was not observed in any other cohort, suggesting the associations were false positives (Supplemental Tables S4 and S5).

One of the key limitations of cohort‐based epigenetic studies is the lack of cell‐sorted data for analysis. As discussed by Birney and colleagues,^30^ optimal planning at the outset of a study is ideal; however, such coordination is difficult to implement in large cohorts and so bioinformatics methods must be applied post hoc to adjust for suboptimal study designs. We adjusted for cell heterogeneity within whole blood, and therefore the signal we tested for association with BMD would be ubiquitous within whole blood. Such ubiquitous signals within whole blood may only be detectable for extremely strong environmental modifiers of DNA methylation, such as cigarette smoking.^5^, ^6^ Targeted EWAS of specific cell types within whole blood with clear roles in bone biology, such as monocytes due to their role in osteoclastogenesis, may be more fruitful. Furthermore, longitudinal studies can identify disease‐risk biomarkers and provide mechanistic insights; however, these are generally underpowered when studying BMD because of the relatively small changes in BMD that occur over time. As we have shown that large‐scale whole blood EWAS of BMD does not identify disease‐risk biomarkers for osteoporosis risk, a well‐powered longitudinal study with a wide range of time points and bone samples may be more informative.

In the largest EWAS meta‐analysis to date of BMD, we observed a probe near *CES1* to be associated with FN BMD in the discovery sample of up to 4826 individuals but not with the same phenotype in a related validation sample of 901 individuals. In conclusion, these findings suggest that there are no large effects of methylation changes on BMD in whole blood in the epigenome, which are common and well captured by the Infinium HumanMethylation450 BeadChip.

## Disclosures

All authors state that they have no conflicts of interest.

## Supporting information

Supporting Data S1.

## References

[1] Burge R , Dawson‐Hughes B , Solomon DH , Wong JB , King A , Tosteson A. Incidence and economic burden of osteoporosis‐related fractures in the United States, 2005‐2025. J Bone Miner Res. 2007; 22(3):465–75. 17144789 10.1359/jbmr.061113

[2] Hernlund E , Svedbom A , Ivergard M , et al. Osteoporosis in the European Union: medical management, epidemiology and economic burden: a report prepared in collaboration with the International Osteoporosis Foundation (IOF) and the European Federation of Pharmaceutical Industry Associations (EFPIA). Arch Osteoporos. 2013; 8(1–2):136. 24113837 10.1007/s11657-013-0136-1 PMC3880487

[3] Estrada K , Styrkarsdottir U , Evangelou E , et al. Genome‐wide meta‐analysis identifies 56 bone mineral density loci and reveals 14 loci associated with risk of fracture. Nat Genet. 2012; 44(5):491–501. 22504420 10.1038/ng.2249 PMC3338864

[4] Zheng H‐F , Forgetta V , Hsu Y‐H , et al. Whole‐genome sequencing identifies EN1 as a determinant of bone density and fracture. Nature. 2015; 526(7571):112–7. 26367794 10.1038/nature14878 PMC4755714

[5] Tsaprouni LG , Yang T‐P , Bell J , et al. Cigarette smoking reduces DNA methylation levels at multiple genomic loci but the effect is partially reversible upon cessation. Epigenetics. 2014; 9(10):1382–96. 25424692 10.4161/15592294.2014.969637 PMC4623553

[6] Joehanes R , Just AC , Marioni RE , et al. Epigenetic signatures of cigarette smoking. Circ Cardiovasc Genet. 2016;CIRCGENETICS.116.001506. 10.1161/CIRCGENETICS.116.001506 PMC5267325 27651444

[7] Robertson KD. DNA methylation and human disease. Nat Rev Genet. 2005; 6(8):597–610. 16136652 10.1038/nrg1655

[8] Suzuki MM , Bird A. DNA methylation landscapes: provocative insights from epigenomics. Nat Rev Genet. 2008; 9(6):465–76. 18463664 10.1038/nrg2341

[9] Grundberg E , Meduri E , Sandling JK , et al. Global analysis of DNA methylation variation in adipose tissue from twins reveals links to disease‐associated variants in distal regulatory elements. Am J Hum Genet. 2013; 93(5):876–90. 24183450 10.1016/j.ajhg.2013.10.004 PMC3824131

[10] Yuan W , Xia Y , Bell CG , et al. An integrated epigenomic analysis for type 2 diabetes susceptibility loci in monozygotic twins. Nat Commun. 2014; 5:5719. 25502755 10.1038/ncomms6719 PMC4284644

[11] Chambers JC , Loh M , Lehne B , et al. Epigenome‐wide association of DNA methylation markers in peripheral blood from Indian Asians and Europeans with incident type 2 diabetes: a nested case‐control study. Lancet Diabetes Endocrinol. 2015; 3(7):526–34. 26095709 10.1016/S2213-8587(15)00127-8 PMC4724884

[12] Tsai P‐C , Van Dongen J , Tan Q , et al. DNA methylation changes in the IGF1R gene in birth weight discordant adult monozygotic twins. Twin Res Hum Genet. 2015; 18(6):635–46. 26563994 10.1017/thg.2015.76

[13] Reppe S , Noer A , Grimholt RM , et al. Methylation of bone SOST, its mRNA, and serum sclerostin levels correlate strongly with fracture risk in postmenopausal women. J Bone Miner Res. 2015; 30(2):249–56. 25155887 10.1002/jbmr.2342

[14] Delgado‐Calle J , Sañudo C , Bolado A , et al. DNA methylation contributes to the regulation of sclerostin expression in human osteocytes. J Bone Miner Res. 2012; 27(4):926–37. 22162201 10.1002/jbmr.1491

[15] Greenblatt MB , Shim J‐H. Osteoimmunology: a brief introduction. Immune Netw. 2013; 13(4):111–5. 24009537 10.4110/in.2013.13.4.111 PMC3759707

[16] Novack DV , Mbalaviele G. Osteoclasts‐key players in skeletal health and disease. Microbiol Spectr. 2016; 4(3). 10.1128/microbiolspec.MCHD-0011-2015 PMC4920143 27337470

[17] Delgado‐Calle J , Fernández AF , Sainz J , et al. Genome‐wide profiling of bone reveals differentially methylated regions in osteoporosis and osteoarthritis. Arthritis Rheum. 2013; 65(1):197–205. 23124911 10.1002/art.37753

[18] Orozco LD , Morselli M , Rubbi L , et al. Epigenome‐wide association of liver methylation patterns and complex metabolic traits in mice. Cell Metab. 2015; 21(6):905–17. 26039453 10.1016/j.cmet.2015.04.025 PMC4454894

[19] Christiansen L , Lenart A , Tan Q , et al. DNA methylation age is associated with mortality in a longitudinal Danish twin study. Aging Cell. 2016; 15(1):149–54. 26594032 10.1111/acel.12421 PMC4717264

[20] Relton CL , Gaunt T , McArdle W , et al. Data resource profile: Accessible Resource for Integrated Epigenomic Studies (ARIES). Int J Epidemiol. 2015; 44(4):1181–90. 25991711 10.1093/ije/dyv072 PMC5593097

[21] Houseman EA , Accomando WP , Koestler DC , et al. DNA methylation arrays as surrogate measures of cell mixture distribution. BMC Bioinformatics. 2012; 13(1):86. 22568884 10.1186/1471-2105-13-86 PMC3532182

[22] Willer CJ , Li Y , Abecasis GR. METAL: fast and efficient meta‐analysis of genomewide association scans. Bioinformatics. 2010; 26(17):2190–1. 20616382 10.1093/bioinformatics/btq340 PMC2922887

[23] Boker S , Neale M , Maes H , et al. OpenMx: an open source extended structural equation modeling framework. Psychometrika. 2011; 76(2):306–17. 23258944 10.1007/s11336-010-9200-6 PMC3525063

[24] Sherry ST , Ward MH , Kholodov M , et al. dbSNP: the NCBI database of genetic variation. Nucleic Acids Res. 2001; 29(1):308–11. 11125122 10.1093/nar/29.1.308 PMC29783

[25] GTEx Consortium . Human genomics. The Genotype‐Tissue Expression (GTEx) pilot analysis: multitissue gene regulation in humans. Science. 2015; 348(6235):648–60. 25954001 10.1126/science.1262110 PMC4547484

[26] Welter D , MacArthur J , Morales J , et al. The NHGRI GWAS Catalog, a curated resource of SNP‐trait associations. Nucleic Acids Res. 2014;42(Database issue):D1001–6. 24316577 10.1093/nar/gkt1229 PMC3965119

[27] Richards JB , Zheng H‐F , Spector TD. Genetics of osteoporosis from genome‐wide association studies: advances and challenges. Nat Rev Genet. 2012; 13(8):576–88. 22805710 10.1038/nrg3228

[28] Dick KJ , Nelson CP , Tsaprouni L , et al. DNA methylation and body‐mass index: a genome‐wide analysis. Lancet. 2014; 383(9933):1990–8. 24630777 10.1016/S0140-6736(13)62674-4

[29] Delgado‐Calle J , Sañudo C , Fernández AF , García‐Renedo R , Fraga MF , Riancho JA. Role of DNA methylation in the regulation of the RANKL‐OPG system in human bone. Epigenetics. 2012; 7(1):83–91. 22207352 10.4161/epi.7.1.18753 PMC3337833

[30] Birney E , Smith GD , Greally JM . Epigenome‐wide association studies and the interpretation of disease ‐omics. PLoS Genet. 2016; 12(6):e1006105. 27336614 10.1371/journal.pgen.1006105 PMC4919098

