# Supplementary material for: Epigenome‐wide Association of DNA Methylation in Whole Blood With Bone Mineral Density
Source: J Bone Miner Res. 2017 May 8;32(8):1644–50. doi: 10.1002/jbmr.3148 (PMC5615229; doi:10.1002/jbmr.3148)

**Supplementary Tables**

**Supplementary Table 1.** Cohort descriptions.

| **Abbreviation** | **Cohort Name** | **Cohort Type** | **Country of Origin** | **Ethnicity** | **BMD Measurement Method (Lunar, Hologic, Norland)** | **Short Cohort Description** | **References** |
| --- | --- | --- | --- | --- | --- | --- | --- |
| ALSPAC | The Avon Longitudinal Study of Parents and Children | Population‐based, family‐based | United Kingdom | North‐western European | GE lunar ProdigyTM | ALSPAC (http://www.bris.ac.uk/alspac/researchers/data-access/data-dictionary/) is a long-term health research project. 14,541 mothers enrolled during pregnancy in 1991 and 1992, and the health and development of their children has been followed in great details, Ethical approval for the study was obtained from the ALSPAC Ethics and Law Committee and the Local Research Ethics Committees.  The ALSPAC methylation data was generated as the ARIES project. ARIES is a BBSRC-funded resource of epigenomic information on a range of human tissues, including DNA methylation data on peripheral blood at multiple time points across the life course.  ALSPAC and ARIES project measured BMD and methylation at different time points, in this study, we used mothers’ measures at middle age for both measures | Paternoster L, Lorentzon M, Lehtimäki T, Eriksson J, Kähönen M, Raitakari O, Laaksonen M, Sievänen H, Viikari J, Lyytikäinen LP, Mellström D, Karlsson M, Ljunggren O, Grundberg E, Kemp JP, Sayers A, Nethander M, Evans DM, Vandenput L, Tobias JH, Ohlsson C. Genetic determinants of trabecular and cortical volumetric bone mineral densities and bone microstructure. PLoS Genet. 2013;9(2):e1003247. PMID:23437003.  Gaunt TR, Shihab HA, Hemani G, Min JL, Woodward G, Lyttleton O, Zheng J, Duggirala A, McArdle WL, Ho K, Ring SM, Evans DM, Davey Smith G, Relton CL. Genome Biol. 2016 Mar 31;17:61. PMID: 27036880.  Fraser A, Macdonald-Wallis C, Tilling K, Boyd A, Golding J, Davey Smith G, Henderson J, Macleod J, Molloy L, Ness A, Ring S, Nelson SM, Lawlor DA. Int J Epidemiol. 2013 Feb;42(1):97-110.  Caroline L Relton, Tom Gaunt, Wendy McArdle, Karen Ho, Aparna Duggirala, Hashem Shihab, Geoff Woodward, Oliver Lyttleton, David M Evans, Wolf Reik, Yu-Lee Paul, Gabriella Ficz, Susan E Ozanne, Anil Wipat, Keith Flanagan, Allyson Lister, Bastiaan T Heijmans, Susan M Ring, and George Davey Smith. Data Resource Profile: Accessible Resource for Integrated Epigenomic Studies (ARIES) Int. J. Epidemiol. (2015) 44 (4): 1181-1190 |
| DTR | The Danish Twin Registry | Population‐based, family‐based | Denmark | North‐western European | Hologic Discovery A and GE Lunar Prodigy | The Danish Twin Registry is a nationwide population-based twin registry covering all birth cohorts of Danish twins born since 1870 (including more than 85,000 twin pairs), with several rounds of surveys conducted in the past 60 years and with biological material from approximately 20,000 twins. Bone mineral density was investigated in a survey of birth weight discordant twins collected in 2008-2010 with the aim to investigate the association of birth weight to bone metabolism, diabetes and thyroid function. No overall associations were found. Furthermore, no associations were found between birth weight and single CpGs in these individuals. In the analysis of the present study birth weight was included as a co-variate. | Skytthe A, Christiansen L, Kyvik KO, Bødker FL, Hvidberg L, Petersen I, Nielsen MM, Bingley P, Hjelmborg J, Tan Q, Holm NV, Vaupel JW, McGue M, Christensen K. The Danish Twin Registry: linking surveys, national registers, and biological information. Twin Res Hum Genet. 2013 Feb;16(1):104-11. PMID: 23084092. Frost M, Petersen I, Andersen TL, Langdahl BL, Buhl T, Christiansen L, Brixen K, Christensen K. Birth weight and adult bone metabolism are unrelated: results from birth weight-discordant monozygotic twins. J Bone Miner Res. 2013 Dec;28(12):2561-9. PMID:23703904.  Frost M, Petersen I, Hegedüs L, Christiansen L, Brix T, Christensen K. Regulation of the pituitary-thyroid axis in adulthood is not related to birth weight: evidence from extremely birth weight-discordant monozygotic Danish twin pairs. Thyroid. 2013 Jul;23(7):785-90. PMID:23308389. Frost M, Petersen I, Brixen K, Beck-Nielsen H, Holst JJ, Christiansen L, Højlund K, Christensen K. Adult glucose metabolism in extremely birthweight-discordant monozygotic twins. Diabetologia. 2012 Dec;55(12):3204-12. PMID:22955993. Tan Q, Frost M, Heijmans BT, von Bornemann Hjelmborg J, Tobi EW, Christensen K, Christiansen L. Epigenetic signature of birth weight discordance in adult twins. BMC Genomics. 2014 Dec 4;15:1062. PMID:25476734. |
| FS Gen2 and Gen3 | Framingham Osteoporosis Study | Population‐based, family‐based | United States of America | European American | GE lunar DPX L | The Framingham Osteoporosis Study is an ancillary study of the parent, Framingham Study. The Framingham Study is a family‐based, multigenerational cohort study initiated originally to study the risk factors for cardiovascular disease | Dawber TR, Meadors GF, Moore FE, Jr.: Epidemiological approaches to heart disease: the Framingham Study. Am J Public Health 1951; 41(3):279-286. (PubMed ID Number: 14819398: Splansky GL, Corey D, Yang Q, Atwood LD, Cupples LA, Benjamin EJ, D’Agostino, Sr., RB, Fox CS, Larson MG, Murabito JM, O’Donnell CJ, Vasan RS, Wolf PA, Levy D. The Third Generation Cohort of the National Heart, Lung, and Blood Institute’s Framingham Heart Study: Design, Recruitment, and Initial Examination. Am J Epidemiol 2007;165:1328–1335. Feinleib M, Kannel WB, Garrison RJ, et al. The Framingham Offspring Study. Design and preliminary data. Prev Med 1975;4:518–25. Kiel DP, Demissie S, Dupuis J, Lunetta KL, Murabito JM, Karasik D. Genome-wide association with bone mass and geometry in the Framingham Heart Study. BMC Med Genet 2007;8(Suppl 1):S14 |
| TUK | TwinsUK | Population‐based, family‐based | United Kingdom | North‐western European | Hologic QDR 4500W | TwinsUK is a population‐based registry of British Twins representative of the general British population. | [PMID: 19841454] {Richards, 2009 Collaborative meta‐analysis: associations of 150 candidate genes with osteoporosis and osteoporotic fracture}; [PMID: 18455228] {Richards, 2008 Bone mineral density, osteoporosis, and osteoporotic fractures: a genome‐wide association study} |
| RS | Rotterdam Study | Population‐based, family‐based | The Netherlands | North‐western European | GE lunar ProdigyTM | The Rotterdam Study is a prospective cohort study that started in 1990 in Ommoord, a suburb of Rotterdam, among 10,994, men and women aged 45 and over. | [PMID:26386597] {Hofman, 2015 The Rotterdam Study: 2016 objectives and design update}; |

**Supplementary Table 2.** BMD and Covariate Characteristics for Cohort-Level Data.

|  |  |  | **Males** | | | | | | **Females** | | | | | |
| --- | --- | --- | --- | --- | --- | --- | --- | --- | --- | --- | --- | --- | --- | --- |
| **Short name** | **Trait** | **Measurement Method** | **N** | **Mean** | **SD** | **Median** | **Min** | **Max** | **N** | **Mean** | **SD** | **Median** | **Min** | **Max** |
|  |  |  |  |  |  |  |  |  |  |  |  |  |  |  |
| ALSPAC | age (yrs) | Birth record | . | . | . | . | . | . | 715 | 47.45 | 4.25 | 47.00 | 34.00 | 60.00 |
|  | BMI (kg/m2) | - | . | . | . | . | . | . | . | . | . | . | . | . |
|  | Weight (kg) | Scales | . | . | . | . | . | . | 715 | 71.15 | 14.80 | 68.10 | 45.80 | 136.00 |
|  | Height (cm) | Harpenden Stadiometer | . | . | . | . | . | . | . | . | . | . | . | . |
|  | FNBMD (g/cm2) | GE Lunar Prodigy | . | . | . | . | . | . | 715 | 1.00 | 0.13 | 0.99 | 0.68 | 1.56 |
|  | LSBMD (g/cm2) | . | . | . | . | . | . | . | . | . | . | . | . | . |
|  |  |  |  |  |  |  |  |  |  |  |  |  |  |  |
| DTR | age (yrs) | Age at exam date (birth dates were archived from the Danish Civil Registration System) | 135 | 47 | 15.0 | 36 | 30 | 70 | 132 | 49 | 15.5 | 57 | 30 | 74 |
|  | BMI (kg/m2) | Calculated | 135 | 26.7 | 3.9 | 26.3 | 19.2 | 37.7 | 132 | 24.0 | 4.3 | 23.3 | 17.3 | 38.6 |
|  | Weight (kg) | Standard scale (SECA, Hamburg, Germany) | 135 | 86 | 13.8 | 85 | 58 | 145 | 132 | 66 | 11.0 | 64 | 43 | 111 |
|  | Height (cm) | Harpenden Stadiometer | 135 | 179 | 7.2 | 178 | 165 | 205 | 132 | 166 | 6.4 | 165 | 151 | 181 |
|  | FNBMD (g/cm2) | Hologic Discovery A and GE Lunar Prodigy | 135 | 0.90 | 0.17 | 0.87 | 0.59 | 1.42 | 132 | 0.83 | 0.16 | 0.82 | 0.34 | 1.33 |
|  | LSBMD (g/cm2) | Hologic Discovery A and GE Lunar Prodigy | 128 | 1.08 | 0.15 | 1.08 | 0.72 | 1.52 | 132 | 0.97 | 0.14 | 0.98 | 0.64 | 1.30 |
|  |  |  |  |  |  |  |  |  |  |  |  |  |  |  |
| RS | age (yrs) | Age at exam date | 294 | 59.19 | 7.90 | 59.81 | 47.51 | 84.50 | 356 | 60.83 | 8.24 | 59.79 | 47.70 | 90.09 |
|  | BMI (kg/m2) | Calculated | 294 | 27.42 | 3.61 | 26.77 | 18.35 | 41.91 | 356 | 27.48 | 5.01 | 26.62 | 18.43 | 50.21 |
|  | Weight (kg) | Standard scale | 294 | 87.74 | 13.42 | 85.85 | 54.30 | 145.40 | 356 | 73.67 | 13.73 | 71.55 | 46.70 | 139.20 |
|  | Height (cm) | Stadiometer | 294 | 178.74 | 6.92 | 178.60 | 154.60 | 207.00 | 356 | 163.80 | 6.35 | 164.20 | 145.00 | 188.20 |
|  | FNBMD (g/cm2) | GE-lunar Prodigy | 294 | 0.97 | 0.13 | 0.96 | 0.64 | 1.35 | 356 | 0.89 | 0.13 | 0.88 | 0.57 | 1.27 |
|  | LSBMD (g/cm2) | GE-lunar Prodigy | 287 | 1.17 | 0.17 | 0.15 | 0.78 | 1.70 | 346 | 1.07 | 0.20 | 1.06 | 0.59 | 1.62 |
|  |  |  |  |  |  |  |  |  |  |  |  |  |  |  |
| TUK | age (yrs) | Age at exam date | . | . | . | . | . | . | 775 | 57.95 | 10.33 | 58.80 | 18.65 | 83.14 |
|  | BMI (kg/m2) | Calculated | . | . | . | . | . | . | 775 | 26.79 | 5.09 | 24.84 | 16.60 | 52.71 |
|  | Weight (kg) | Standard scale | . | . | . | . | . | . | 775 | 69.09 | 13.02 | 67.40 | 37.90 | 126.80 |
|  | Height (cm) | Stadiometer | . | . | . | . | . | . | 775 | 160.7 | 6.58 | 161.0 | 143.0 | 181.0 |
|  | FNBMD (g/cm2) | Hologic QDR 4500W | . | . | . | . | . | . | 775 | 0.78 | 0.13 | 0.77 | 0.47 | 1.17 |
|  | LSBMD (g/cm2) | Hologic QDR 4500W | . | . | . | . | . | . | 770 | 0.97 | 0.15 | 0.97 | 0.59 | 1.43 |

**Supplementary Table 3.** Metrics for DNA Methylation Quantification for Each Cohort.

| **Cohort** | **Platform** | **QC and Probe Normalization** | **Additional QC filters pre-association** |
| --- | --- | --- | --- |
|  |  |  |  |
| ALSPAC | Illumina HumanMethylation450 Bead Chip | Methylation data for mothers at middle age were used in this study. Excluded probes with detection P-value > 0.01 in more than 5% of samples. Excluded samples if less than 95% of probes have detection P-values < 0.01. Used BMIQ for probe normalization. | Genotype probes on the 450 K BeadChip were compared between samples from the same individual and against SNP-chip data to identify and remove any sample mismatches |
| DTR | Illumina HumanMethylation450 Bead Chip | Excluded probes with detection P-value > 0.01 in more than 5% of samples. Excluded samples if less than 95% of probes have detection P-values < 0.01. Used BMIQ for probe normalization. | Sample exclusion: the default criteria for the internal quality control probes of the bead chip were applied in the MethylAid package in R (PMID:25944819, PMID:25147358) for identifying low quality samples. Probe exclusion: a raw intensity value of zero, bead counts per probe < 3, detection P-value > 0.01, as well as probes identified as being cross reactive (PMID: 23314698). Subsequent probe and sample success rates <0.95. |
| FOS | Illumina HumanMethylation450 Bead Chip | Excluded probes with detection P-value > 0.01 in more than 5% of samples. Excluded samples if less than 95% of probes have detection P-values < 0.01. Used DASEN for probe normalization. |  |
| FOS Gen3 | Illumina HumanMethylation450 Bead Chip | Excluded probes with detection P-value > 0.01 in more than 5% of samples. Excluded samples if less than 95% of probes have detection P-values < 0.01. Used DASEN for probe normalization. |  |
| RS | Illumina HumanMethylation450 Bead Chip | Excluded probes with detection P-value > 0.01 in more than 1% of samples, as well as probes with less than 4 informative beads. Samples were excluded when showing a low detection rate (<99%), incomplete bisulfite conversion, or gender swaps. Used DASEN for probe normalization. |  |
| TUK | Illumina HumanMethylation450 Bead Chip | Excluded probes with detection P-value > 0.01 in more than 5% of samples. Excluded samples if less than 95% of probes have detection P-values < 0.01. Used BMIQ for probe normalization. |  |

**Supplementary Table 4.** Cohort-Level Significant Results for Association Testing.

| **BMD Trait** | **Cohort** | **Analysis** | **Probe** | **Effect Size** | **Standard Error** | **P-Value** | **P-Value, BH-Adjusted** | **Sample Size** |
| --- | --- | --- | --- | --- | --- | --- | --- | --- |
|  |  |  |  |  |  |  |  |  |
| **LS** | **DTR** | **Female** | cg04081651 | 3.69 | 0.63 | 4.38E-07 | 4.44E-02 | 132 |
|  |  |  | cg09832237 | -3.77 | 0.64 | 5.58E-08 | 2.26E-02 | 132 |
|  |  |  | cg14793931 | 3.71 | 0.69 | 3.94E-07 | 4.44E-02 | 132 |
|  |  |  | cg24029028 | -3.49 | 0.61 | 3.32E-07 | 4.44E-02 | 131 |
|  |  | **Male** | cg23214071 | 2.1 | 0.36 | 1.05E-07 | 4.26E-02 | 128 |
|  | **TUK** | **Female** | cg24117468 | 1.46 | 0.25 | 1.09E-08 | 3.40E-03 | 770 |
|  |  |  | cg02526790 | -0.25 | 0.24 | 1.68E-08 | 3.40E-03 | 770 |

**Supplementary Table 5.** Cohort-Specific Results for Four Probes Significant in DTR for Female LS BMD Analyses.

| Probe | Study | BMD Trait | Analysis | Effect Size | Standard Error | P-Value | Sample Size |
| --- | --- | --- | --- | --- | --- | --- | --- |
| cg04081651 | ALSPAC | FN | FEMALE | 0.37 | 0.30 | 0.21 | 927 |
|  | DTR | FN | FEMALE | 1.58 | 0.64 | 0.02 | 132 |
|  |  |  | POOLED | 0.47 | 0.44 | 0.30 | 267 |
|  |  | LS | FEMALE | 3.69 | 0.63 | 4.38E-07 | 132 |
|  |  |  | POOLED | 0.80 | 0.47 | 0.09 | 260 |
|  | FOS | FN | FEMALE | -0.04 | 0.22 | 0.86 | 1254 |
|  |  |  | POOLED | 0.22 | 0.15 | 0.15 | 2207 |
|  |  | LS | FEMALE | 0.12 | 0.15 | 0.42 | 1259 |
|  |  |  | POOLED | 0.18 | 0.10 | 0.06 | 2203 |
|  | RS | FN | FEMALE | 0.78 | 0.39 | 0.04 | 356 |
|  |  |  | POOLED | 0.85 | 0.27 | 2.09E-03 | 650 |
|  |  | LS | FEMALE | 0.49 | 0.26 | 0.06 | 346 |
|  |  |  | POOLED | 0.26 | 0.20 | 0.19 | 633 |
|  | TUK | FN | FEMALE | -0.20 | 0.22 | 0.36 | 775 |
|  |  | LS | FEMALE | 0.06 | 0.17 | 0.75 | 770 |
| cg09832237 | ALSPAC | FN | FEMALE | -0.21 | 0.30 | 0.49 | 927 |
|  | DTR | FN | FEMALE | -0.93 | 0.64 | 0.15 | 132 |
|  |  |  | POOLED | -0.53 | 0.43 | 0.22 | 267 |
|  |  | LS | FEMALE | -3.77 | 0.64 | 5.58E-08 | 132 |
|  |  |  | POOLED | -1.94 | 0.45 | 3.10E-05 | 260 |
|  | FOS | FN | FEMALE | 0.21 | 0.24 | 0.39 | 1254 |
|  |  |  | POOLED | 0.00 | 0.17 | 0.98 | 2207 |
|  |  | LS | FEMALE | 0.21 | 0.16 | 0.18 | 1259 |
|  |  |  | POOLED | 0.02 | 0.11 | 0.83 | 2203 |
|  | RS | FN | FEMALE | -0.36 | 0.44 | 0.41 | 356 |
|  |  |  | POOLED | -0.67 | 0.30 | 0.03 | 650 |
|  |  | LS | FEMALE | -0.41 | 0.30 | 0.16 | 346 |
|  |  |  | POOLED | -0.68 | 0.21 | 1.51E-03 | 633 |
|  | TUK | FN | FEMALE | 0.08 | 0.29 | 0.79 | 775 |
|  |  | LS | FEMALE | 0.00 | 0.23 | 1.00 | 770 |
| cg14793931 | ALSPAC | FN | FEMALE | 0.69 | 0.30 | 0.02 | 927 |
|  | DTR | FN | FEMALE | 1.76 | 0.67 | 0.01 | 132 |
|  |  |  | POOLED | 0.10 | 0.47 | 0.82 | 267 |
|  |  | LS | FEMALE | 3.71 | 0.69 | 3.94E-07 | 132 |
|  |  |  | POOLED | 0.85 | 0.49 | 0.09 | 260 |
|  | FOS | FN | FEMALE | -0.03 | 0.24 | 0.91 | 1254 |
|  |  |  | POOLED | -0.08 | 0.17 | 0.66 | 2207 |
|  |  | LS | FEMALE | 0.14 | 0.16 | 0.37 | 1259 |
|  |  |  | POOLED | 0.12 | 0.11 | 0.30 | 2203 |
|  | RS | FN | FEMALE | -0.14 | 0.40 | 0.72 | 356 |
|  |  |  | POOLED | -0.47 | 0.28 | 0.09 | 650 |
|  |  | LS | FEMALE | -0.06 | 0.28 | 0.83 | 346 |
|  |  |  | POOLED | -0.16 | 0.20 | 0.41 | 633 |
|  | TUK | FN | FEMALE | 0.03 | 0.31 | 0.93 | 775 |
|  |  | LS | FEMALE | -0.02 | 0.24 | 0.93 | 770 |
| cg24029028 | ALSPAC | FN | FEMALE | -0.49 | 0.30 | 0.10 | 927 |
|  | DTR | FN | FEMALE | -2.35 | 0.57 | 1.50E-04 | 131 |
|  |  |  | POOLED | -1.22 | 0.40 | 2.41E-03 | 265 |
|  |  | LS | FEMALE | -3.49 | 0.61 | 3.32E-07 | 131 |
|  |  |  | POOLED | -1.75 | 0.41 | 3.61E-05 | 258 |
|  | FOS | FN | FEMALE | 0.22 | 0.20 | 0.27 | 1254 |
|  |  |  | POOLED | -0.01 | 0.12 | 0.94 | 2207 |
|  |  | LS | FEMALE | 0.08 | 0.13 | 0.52 | 1259 |
|  |  |  | POOLED | -0.09 | 0.08 | 0.28 | 2203 |
|  | RS* | FN | FEMALE | NA | NA | NA | 356 |
|  |  |  | POOLED | NA | NA | NA | 650 |
|  |  | LS | FEMALE | NA | NA | NA | 346 |
|  |  |  | POOLED | NA | NA | NA | 633 |
|  | TUK | FN | FEMALE | -0.28 | 0.32 | 0.38 | 775 |
|  |  | LS | FEMALE | -0.31 | 0.25 | 0.20 | 770 |

*cg24029028 was removed from RS due at the quality control stage

**Supplementary Table 6.** Cohort-Specific Results for cg23214071, the Probe Significant in DTR for Male LS BMD Analyses. Only cohorts with male samples are shown.

| Study | BMD Trait | Analysis | Effect Size | Standard Error | P-Value | Sample Size |
| --- | --- | --- | --- | --- | --- | --- |
| DTR | FN | MALE | 1.09 | 0.38 | 0.01 | 135 |
|  |  | POOLED | 0.78 | 0.27 | 4.48E-03 | 267 |
|  | LS | MALE | 2.10 | 0.36 | 1.05E-07 | 128 |
|  |  | POOLED | 1.36 | 0.28 | 2.16E-06 | 260 |
| FOS | FN | MALE | -0.26 | 0.26 | 0.32 | 953 |
|  |  | POOLED | -0.31 | 0.18 | 0.08 | 2207 |
|  | LS | MALE | -0.12 | 0.17 | 0.47 | 953 |
|  |  | POOLED | -0.27 | 0.11 | 0.02 | 2203 |
| RS | FN | MALE | 0.56 | 0.44 | 0.21 | 294 |
|  |  | POOLED | 0.20 | 0.31 | 0.52 | 650 |
|  | LS | MALE | 0.31 | 0.33 | 0.36 | 287 |
|  |  | POOLED | -0.16 | 0.22 | 0.46 | 633 |

**Supplementary Table 7.** Cohort-Specific Results for Two Probes Significant in TUK for Female LS BMD Analyses.

| **Probe** | **Study** | **BMD Trait** | **Analysis** | **Effect Size** | **Standard Error** | **P-Value** | **Sample Size** |
| --- | --- | --- | --- | --- | --- | --- | --- |
| cg24117468 | ALSPAC | FN | FEMALE | 0.55 | 0.3 | 0.07 | 927 |
|  | DTR | FN | FEMALE | 0.56 | 0.6 | 0.37 | 132 |
|  |  |  | POOLED | 0.04 | 0.43 | 0.93 | 267 |
|  |  | LS | FEMALE | 0.18 | 0.67 | 0.79 | 132 |
|  |  |  | POOLED | 0 | 0.46 | 0.99 | 260 |
|  | FOS | FN | FEMALE | -0.17 | 0.23 | 0.48 | 1254 |
|  |  |  | POOLED | 0.06 | 0.17 | 0.72 | 2207 |
|  |  | LS | FEMALE | -0.22 | 0.16 | 0.17 | 1259 |
|  |  |  | POOLED | 0.1 | 0.11 | 0.35 | 2203 |
|  | RS | FN | FEMALE | -0.36 | 0.42 | 0.4 | 356 |
|  |  |  | POOLED | -0.01 | 0.29 | 0.97 | 650 |
|  |  | LS | FEMALE | 0.21 | 0.29 | 0.48 | 346 |
|  |  |  | POOLED | 0.15 | 0.2 | 0.45 | 633 |
|  | TUK | FN | FEMALE | 0.86 | 0.32 | 8.19E-03 | 775 |
|  |  | LS | FEMALE | 1.46 | 0.25 | 1.09E-08 | 770 |
| cg02526790 | ALSPAC | FN | FEMALE | -0.21 | 0.3 | 0.48 | 927 |
|  | DTR | FN | FEMALE | 0.27 | 0.62 | 0.66 | 132 |
|  |  |  | POOLED | -0.22 | 0.44 | 0.61 | 267 |
|  |  | LS | FEMALE | -0.42 | 0.7 | 0.56 | 132 |
|  |  |  | POOLED | -0.59 | 0.47 | 0.21 | 260 |
|  | FOS | FN | FEMALE | -0.18 | 0.24 | 0.44 | 1254 |
|  |  |  | POOLED | -0.12 | 0.17 | 0.49 | 2207 |
|  |  | LS | FEMALE | 0.13 | 0.16 | 0.41 | 1259 |
|  |  |  | POOLED | 0.12 | 0.11 | 0.27 | 2203 |
|  | RS | FN | FEMALE | -0.61 | 0.45 | 0.18 | 356 |
|  |  |  | POOLED | -0.32 | 0.31 | 0.3 | 650 |
|  |  | LS | FEMALE | -0.11 | 0.31 | 0.73 | 346 |
|  |  |  | POOLED | -0.24 | 0.22 | 0.27 | 633 |
|  | TUK | FN | FEMALE | -0.03 | 0.31 | 0.92 | 775 |
|  |  | LS | FEMALE | -0.25 | 0.24 | 1.68E-08 | 770 |

**Supplementary Table 8.** Cohort-Specific Results for cg23196985, the Probe Significant in Discovery FN Female and Sex-Pooled Meta-Analyses.

| **BMD Trait** | **Analysis** | **Study** | **Effect Size** | **Standard Error** | **P-Value** | **Sample Size** |
| --- | --- | --- | --- | --- | --- | --- |
| FN | FEMALE | ALSPAC* | 1.03 | 0.3 | 5.68E-04 | 927 |
|  |  | DTR | 0.91 | 0.51 | 7.94E-02 | 132 |
|  |  | FOS | 0.79 | 0.24 | 1.26E-03 | 1254 |
|  |  | RS | 0.68 | 0.46 | 1.35E-01 | 356 |
|  |  | TUK* | 1.2 | 0.27 | 1.14E-05 | 775 |
|  | MALE | DTR | 0.17 | 0.46 | 7.08E-01 | 135 |
|  |  | FOS | 0.19 | 0.26 | 4.72E-01 | 953 |
|  |  | RS | 0.14 | 0.46 | 7.57E-01 | 294 |
|  | POOLED | DTR | 0.27 | 0.34 | 4.36E-01 | 267 |
|  |  | FOS | 0.48 | 0.17 | 6.06E-03 | 2207 |
|  |  | RS | 0.44 | 0.32 | 1.74E-01 | 650 |
| LS | FEMALE | DTR | 1.22 | 0.57 | 4.21E-02 | 132 |
|  |  | FOS | 0.36 | 0.16 | 2.92E-02 | 1259 |
|  |  | RS | 0.47 | 0.31 | 1.29E-01 | 346 |
|  |  | TUK* | 0.24 | 0.22 | 2.72E-01 | 770 |
|  | MALE | DTR | 0.2 | 0.48 | 6.69E-01 | 128 |
|  |  | FOS | 0.01 | 0.17 | 9.49E-01 | 953 |
|  |  | RS | 0.04 | 0.35 | 9.07E-01 | 287 |
|  | POOLED | DTR | 0.38 | 0.36 | 2.97E-01 | 260 |
|  |  | FOS | 0.17 | 0.11 | 1.27E-01 | 2203 |
|  |  | RS | 0.2 | 0.23 | 3.70E-01 | 633 |
| *Included in pooled analyses because samples were female-only | | | | |  |  |

**Supplementary Table 9.** Genomic Inflation Lambda Values for Each Cohort-Level and Discovery Meta-Analysis.

| **Cohort** | **BMD Trait** | **Analysis** | **Lambda** |
| --- | --- | --- | --- |
| **ALSPAC** | FN | FEMALE | 0.97 |
| DTR | FN | FEMALE | 1.18 |
|  |  | MALE | 1.06 |
|  |  | POOLED | 1.01 |
|  | LS | FEMALE | 1.46 |
|  |  | MALE | 0.99 |
|  |  | POOLED | 1.04 |
| FOS | FN | FEMALE | 1.03 |
|  |  | MALE | 0.91 |
|  |  | POOLED | 1.04 |
|  | LS | FEMALE | 1.08 |
|  |  | MALE | 0.91 |
|  |  | POOLED | 1.02 |
| RS | FN | FEMALE | 1.02 |
|  |  | MALE | 1.18 |
|  |  | POOLED | 1.12 |
|  | LS | FEMALE | 1.12 |
|  |  | MALE | 0.97 |
|  |  | POOLED | 0.99 |
| TUK | FN | FEMALE | 1.15 |
|  | LS | FEMALE | 1 |
| **Meta-Analysis** | **FN** | **FEMALE** | **1.02** |
|  |  | **MALE** | **0.96** |
|  |  | **POOLED** | **0.97** |
|  | **LS** | **FEMALE** | **0.95** |
|  |  | **MALE** | **0.91** |
|  |  | **POOLED** | **0.97** |

**Supplementary Table 10.** Cohort-Level Analyses Between cg23196985 and FN BMD in Females-Only, Adjusted for Four SNPs Mapping to the 50 Base Pair Probe Body.

| **SNP** | **Cohort** | **Minor Allele Frequency** | **Probe-Adjusted P-Value** |
| --- | --- | --- | --- |
| **rs12149371** | ALSPAC | NA | NA |
|  | FOS | 0.147 | 0.55 |
|  | RS | 0.175 | 0.24 |
| **rs12149373** | ALSPAC | NA | NA |
|  | FOS | 0.123 | 0.56 |
|  | RS | 0.19 | 0.37 |
| **rs144950224** | ALSPAC | 0 | NA |
|  | FOS | 0.004 | 0.58 |
|  | RS | 0.001 | 0.13 |
| **rs3815583** | RS | 0.177 | 0.31 |
|  | ALSPAC | 0.096 | 4.16E-04 |
|  | FOS | 0.23 | 0.92 |

**Supplementary Figures**

**Figure Legends**

**Supplementary Figure 1. QQ-plot of the observed –log10 association *P*-values against the expected null distribution, for discovery meta-analysis of FN BMD in males-only.** Genomic inflation lambda values are given to quantify statistical inflation of *P*-values. No evidence for statistical inflation was observed in the QQ-plot or as calculated by the lambda score.

**Supplementary Figure 2. QQ-plots of the observed –log10 association *P*-values against the expected null distribution, for discovery meta-analyses of LS BMD in a) females-only, b) males-only, and c) both females and males.** Genomic inflation lambda values are given to quantify statistical inflation of *P*-values. No evidence for statistical inflation was observed in the QQ-plots or as calculated by lambda scores.

**Supplementary Figure 3. Manhattan plot of –log_10_ association *P*-values for meta-analysis of FN BMD in males-only.**

**Supplementary Figure 4. Manhattan plots of –log_10_ association *P*-values for meta-analyses of LS BMD in a) females-only, b) males-only, and c) both females and males.**

**Figures**

**Supplementary Figure 1.**


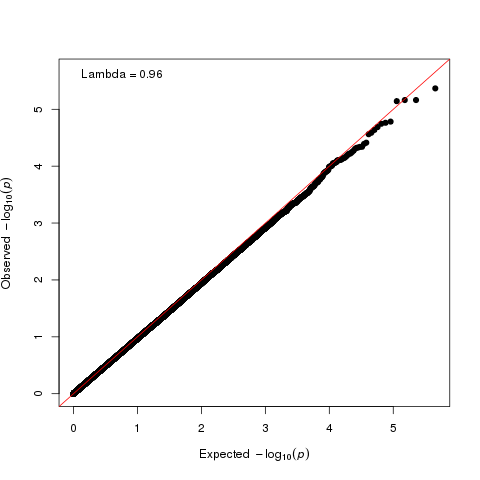


**Supplementary Figure 2. a)**


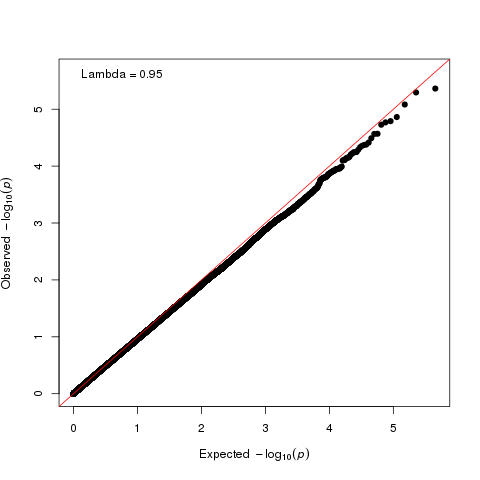


**Supplementary Figure 2. b)**


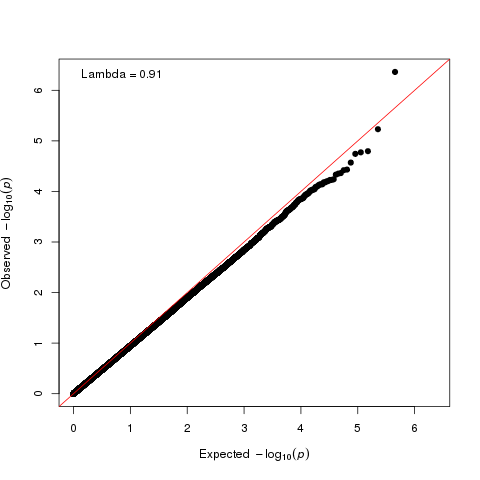


**Supplementary Figure 2. c)**


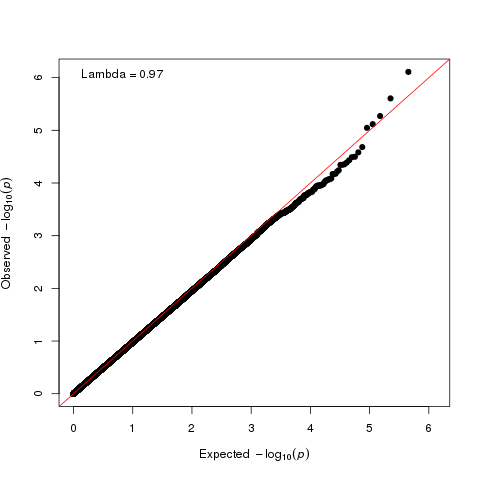


**Supplementary Figure 3.**


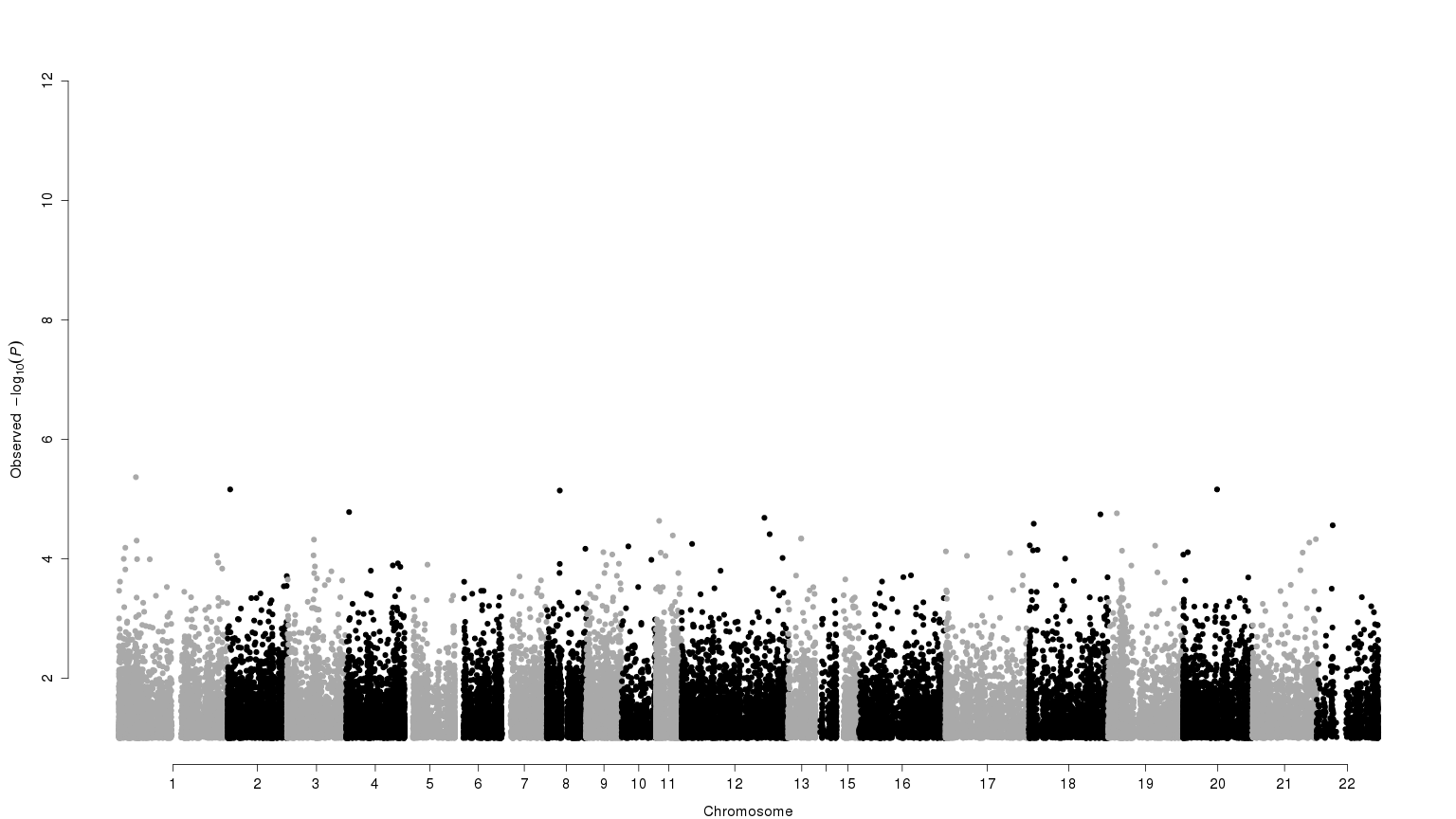


**Supplementary Figure 4. a)**


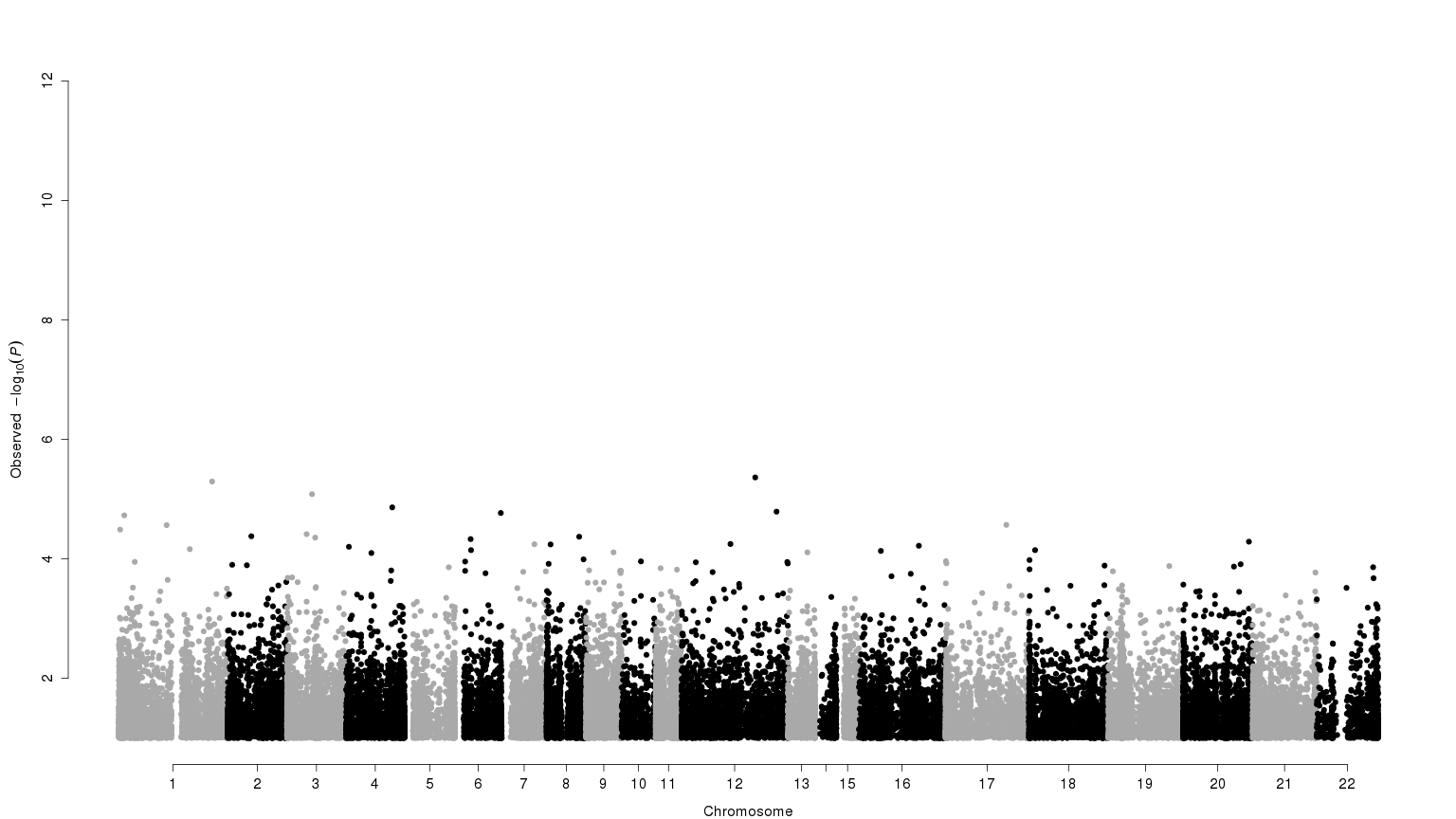


**Supplementary Figure 4. b)**


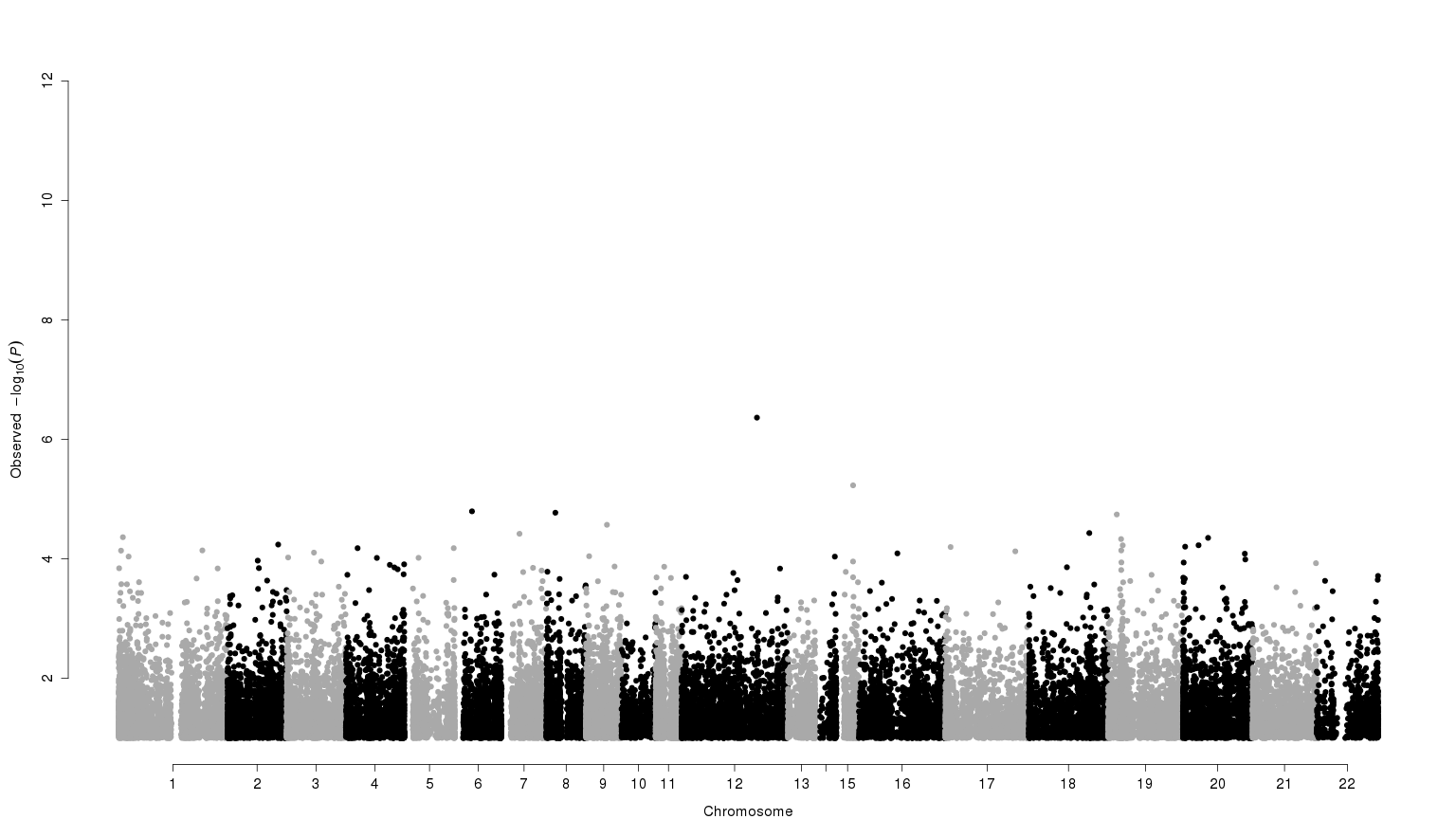


**Supplementary Figure 4. c)**


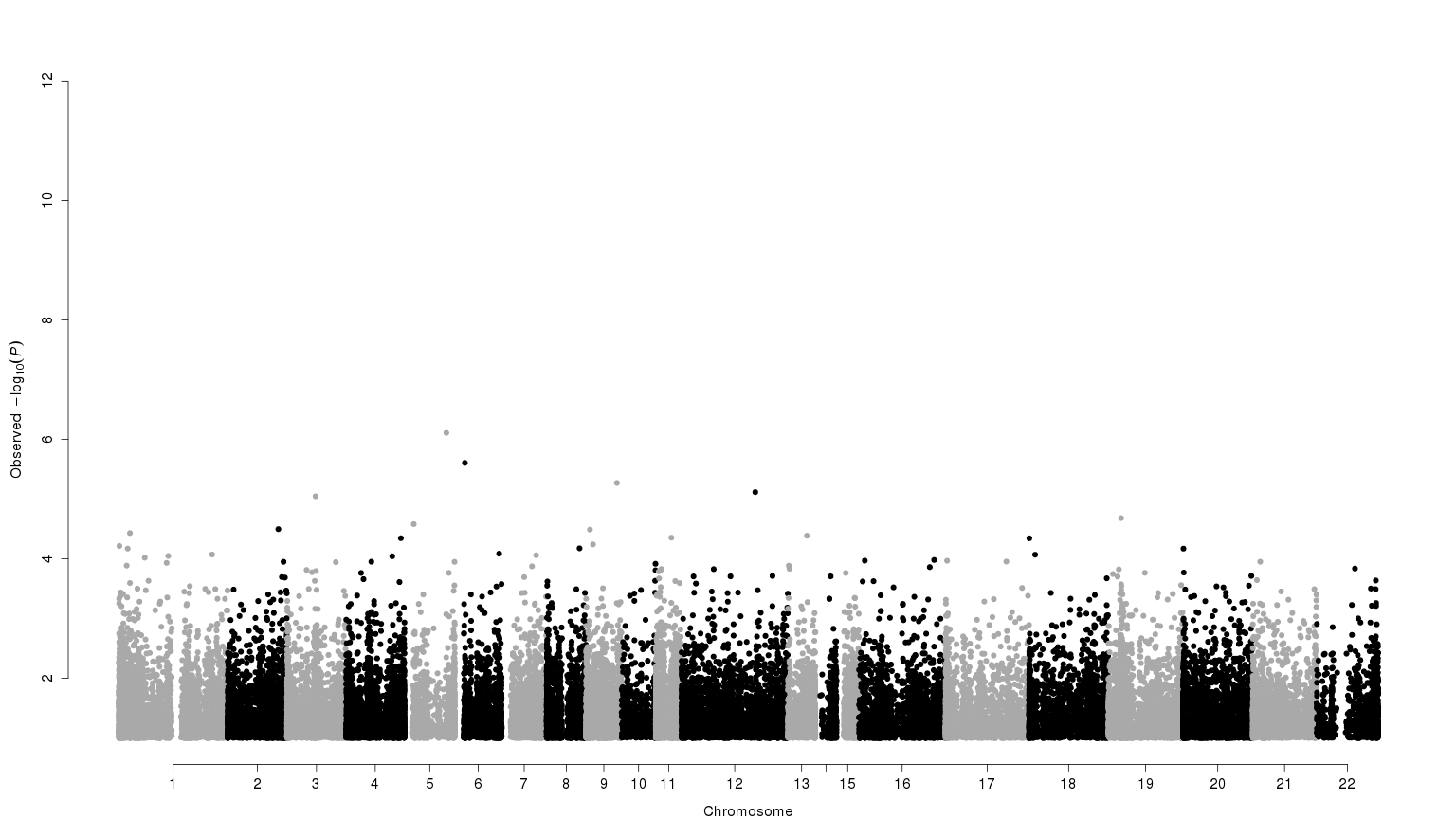

Supplement: Supplementary file 1 — Supporting Data S1. [file JBMR-32-1644-s001.docx]
